# Yokukansan, a Kampo Medicine, Protects PC12 Cells from Glutamate-Induced Death by Augmenting Gene Expression of Cystine/Glutamate Antiporter System Xc^−^

**DOI:** 10.1371/journal.pone.0116275

**Published:** 2014-12-31

**Authors:** Hitomi Kanno, Zenji Kawakami, Kazushige Mizoguchi, Yasushi Ikarashi, Yoshio Kase

**Affiliations:** Tsumura Research Laboratories, Kampo Scientific Strategies Division, Tsumura & Co., Inashiki, Ibaraki, Japan; Hokkaido University, Japan

## Abstract

Effects of the kampo medicine yokukansan on gene expression of the cystine/glutamate antiporter system Xc^−^, which protects against glutamate-induced cytotoxicity, were examined in Pheochromocytoma cells (PC12 cells). Yokukansan inhibited glutamate-induced PC12 cell death. Similar cytoprotective effects were found in Uncaria hook. Experiments to clarify the active compounds revealed that geissoschizine methyl ether, hirsuteine, hirsutine, and procyanidin B1 in Uncaria hook, had cytoprotective effects. These components enhanced gene expressions of system Xc^−^ subunits xCT and 4F2hc, and also ameliorated the glutamate-induced decrease in glutathione levels. These results suggest that the cytoprotective effect of yokukansan may be attributed to geissoschizine methyl ether, hirsuteine, hirsutine, and procyanidin B1 in Uncaria hook.

## Introduction

Glutamate-mediated toxicity is an important mechanism of neuronal death in various pathologic conditions including ischemia [1], trauma [2], epileptic seizures [3], and neurodegenerative disorders such as Alzheimer's, Parkinson's, and Huntington's diseases [4], [5]. To date, two mechanisms have been proposed for glutamate neurotoxicity, i.e., glutamate receptor-mediated neurotoxicity [6], [7] and cystine/glutamate antiporter system Xc^−^ inhibition-mediated neurotoxicity [8]–[11]. Pheochromocytoma cells (PC12 cells) are demonstrated to express system Xc^−^, but do not exhibit the normal NMDA receptor profile [10]–[14]. We have also demonstrated that PC12 cells lack NR2A and NR2B subunits in the NMDA receptor, although system Xc^−^, consisting of xCT and 4F2hc subunits, is expressed in PC12 cells as well as in primary cultured neurons [15]. These findings also suggest that the PC12 cell is a valuable tool for selective evaluation of test substances on system Xc^−^.

Yokukansan (YKS) is one of the traditional Japanese medicines called “*kampo*” medicines in Japan. It is composed of seven kinds of dried medical herbs, and at least 25 ingredients have been identified by three-dimensional chromatography [16]. YKS has been approved by the Ministry of Health, Labour, and Welfare of Japan as a remedy for neurosis, insomnia, and irritability in children. Recently, YKS was reported to improve behavioral and psychological symptoms of dementia such as hallucinations, agitation, and aggressiveness in patients with Alzheimer's disease, dementia with Lewy bodies, vascular dementia, and other forms of senile dementia [16]–[20].

We previously reported that YKS has pharmacologic actions such as glutamate transport activation [21], [22] and cellular protection [15], [23] against glutamate neurotoxicity, suggesting that YKS possesses cytoprotective effect. In those studies, we demonstrated that YKS protected against glutamate-induced PC12 cell death and ameliorated the decrease in glutathione (GSH) levels induced by glutamate [15]. This finding suggested that the protective effect of YKS might be related to system Xc^−^ because a decrease in GSH is known to be induced by inhibition of system Xc^−^. However, the effects of YKS on system Xc^−^ remain unclear.

To clarify this issue, in the present study, we examined the effects of YKS and its constituent herbs and components on glutamate-induced cell death, system Xc^−^ expression, and the GSH level using PC12 cells.

## Materials and Methods

### 1. Drugs and Reagents

#### 1.1. YKS and seven constituent herbs

The dry powdered extracts of YKS and its seven constituent medicinal herbs used in the present study were supplied by Tsumura & Co. (Tokyo, Japan). YKS is composed of seven dried medicinal herbs: 19.5% Atractylodes Lancea rhizome (ALR; rhizome of *Atractylodes lancea* De Candolle, Compositae), 19.5% Poria sclerotium (PS; sclerotium of *Poria cocos* Wolf, Polyporaceae), 14.6% Cnidium rhizome (CR; rhizome of *Cnidium officinale* Makino, Umbelliferae), 14.6% Japanese Angelica root (JAR; root of *Angelica acutiloba* Kitagawa, Umbelliferae), 9.8% Bupleurum root (BR; root of *Bupleurum falcatum* Linné, Umbelliferae), 7.3% Glycyrrhiza (GR; root and stolon of *Glycyrrhiza uralensis* Fisher, Leguminosae), and 14.6% Uncaria hook (UH; hook of *Uncaria rhynchophilla* Miquel, Rubiaceae). The seven medical herbs were extracted with purified water at 95°C for 1 h, and the extraction solution was separated from the insoluble waste and concentrated by removing water under reduced pressure. Spray-drying was used to produce a dried extract powder.

We have already reported the three-dimensional high-performance liquid chromatographic analysis of the ingredients of a YKS extract [16]. In brief, the dried extract (1.0 g) of YKS was dissolved in 20 mL methanol under ultrasonication for 30 min and then centrifuged at 3,000 rpm for 5 min. The supernatant was filtered through a membrane with 0.45 µm pores. An aliquot (30 µL) of the filtrate was injected into a high-performance liquid chromatograph (Shimadzu SPD-M10AVP, Shimadzu Co., Kyoto, Japan). At least 25 compounds were identified in the three-dimensional chromatogram [16].

#### 1.2. GR, UH, and ALR components

Eight GR-derived components including liquiritinapioside (LQA), liquiritin (LQ), liquiritigenin (LQG), isoliquiritin (ILQ), isoliquiritigenin (ILQG), glycyrrhizin (GL), 18β-glycyrrhetinic acid (GA), and glycycoumarin (GC), eight UH-derived components including rhynchophylline (RP), isorhynchophylline (IRP), corynoxeine (CX), isocorynoxeine (ICX), geissoschizine methyl ether (GM), hirsuteine (HTE), hirsutine (HIR), and procyanidin B1 (PCB1), and an ALR-derived component, β-eudesmol (EM), were supplied by the Botanical Raw Materials Research Department of Tsumura & Co. (Ibaraki, Japan).

#### 1.3. Reagents for PC12 cell culture, MTT, and GSH assays

Roswell Park Memorial Institute (RPMI)-1640 medium, heat-inactivated horse serum, dialyzed fetal bovine serum, penicillin, and streptomycin were purchased from Invitrogen (Grand Island, NY, USA). Fetal bovine serum was purchased from ICN Biomedicals (Aurora, OH, USA). Dialyzed horse serum was purchased from Tissue Culture Biologicals (Tulare, CA, USA). Glutamate, cystine, sodium dodecyl sulfate (SDS), metaphosphoric acid (MPA), triethanolamine (TEAM), and bovine serum albumin were purchased from Sigma-Aldrich (St. Louis, MO, USA), and 3-(4,5-dimethylthiazol-2-yl)-2,5-diphenyltetrazolium bromide (MTT) was purchased from Dojindo (Kumamoto, Japan). (S)-4-Carboxyphenylglycine (CPG) was purchased from Tocris Bioscience (Bristol, UK). Other chemicals were purchased from commercial sources.

#### 1.4. Reagents for real-time reverse-transcription polymerase chain reaction (real-time RT-PCR) analysis

A Qiagen RNeasy mini kit was purchased from Qiagen (Hilden, Germany). High Capacity cDNA RT Kits, TaqMan Gene Expression Master Mix, and TaqMan gene expression assay probes for detection of xCT (probe ID: Rn01495125_m1), 4F2hc (probe ID: Rn01759900_g1), and GAPDH (probe ID: Rn01775763_g1) were purchased from Applied Biosystems (Foster City, CA, USA).

### 2. PC12 cell preparation

PC12 cells obtained from Dainippon Sumitomo Pharma (Osaka, Japan) were maintained at 37°C in 95% air and 5% CO_2_ with 95% relative humidity in RPMI-1640 medium supplemented with 5% fetal calf serum, 10% heat-inactivated horse serum, 50 U/mL penicillin, and 50 µg/mL streptomycin until used for the experiments on cell death and gene expression of system Xc^−^ and GSH.

### 3. Determination of glutamate-induced PC12 cell death

PC12 cells were seeded into 96-well microplates (50,000 cells/mL medium) and maintained in RPMI-1640 medium supplemented with 5% dialyzed fetal calf serum, 10% dialyzed horse serum, 50 U/mL penicillin, and 50 µg/mL streptomycin. The cultures were incubated for 48 h at 37°C in 95% relative humidity with a mixture of 5% CO_2_ and 95% air. In order to examine the effects of test substances on glutamate-induced cell death, the media were replaced with fresh media including 2.5 mM glutamate or glutamate plus various concentrations of test substances. The medium without glutamate was used as control. After incubation for 24 h, the cell survival rates were evaluated by an MTT reduction assay.

The MTT reduction assay [24] was performed as follows: 20 µL of 5 mg/mL MTT dissolved in phosphate-buffered saline, PBS(−), was added to each well and incubated at 37°C for 5 h. The reaction was stopped by the addition of 100 µL of a solubilization solution (10% SDS in 0.01 N HCl), and the blue formazan formed from MTT by the reaction was dissolved by additional incubation for 18 h at 37°C. Absorbance of the formazan solution was measured using a microplate reader at a test wavelength of 540 nm and a reference wavelength of 690 nm.

The specific MTT absorbance of a test substance-treated sample (OD_test_) or control sample (OD_control_) was defined by subtracting the corresponding blank absorbance (OD_BL_) of the test substance or control solution. Each OD_BL_ was determined in the experimental condition without cultured cells. Cell survival (%) in test substance against control was calculated by the following formula: cell survival (%) = [(OD_test_−OD_test BL_)/(OD_control_−OD_control BL_)]×100.

### 4. Real-time RT-PCR analysis for gene expression of system Xc^−^

PC12 cells were seeded into 6-well microplates (50,000 cells/mL medium) in RPMI-1640 supplemented as described above. The cultures were incubated for 48 h at 37°C in 95% relative humidity with a mixture of 5% CO_2_ and 95% air. In order to examine the effects of test substances on glutamate-induced changes in system Xc^−^ subunits (xCT and 4F2hc), the media were replaced with fresh media including 2.5 mM glutamate or glutamate plus various concentrations of test substances. The medium without glutamate was used as a control. After incubation for 6 h, total RNA was isolated using the Qiagen RNeasy mini kit according to the manufacturer's protocol. In brief, the cultured cells were lysed in RLT buffer containing guanidine-thiocyanate. The lysate was mixed with the same volume of 70% ethanol. The mixture was infused into an RNeasy Mini spin column for adsorption of total RNA. The total RNA adsorbed in the column was finally eluted with 50 µL of RNase-free water. The RNA concentration was determined spectrophotometrically at 260 nm.

Reverse transcription was carried out using High Capacity cDNA RT Kits (Applied Biosystems) according to the manufacturer's protocol with total RNA samples and run on a TAK-TP400 Thermal Cycler (Takara, Shiga, Japan). In brief, 2,500 ng of total RNA was added to the final reaction mixture (50 µL) containing 1× TaqMan RT buffer, 8 mmol/L dNTP mix, 2.5 µmol/L random hexamers, 0.4 U/µL RNase inhibitor, and Multiscribe Reverse Transcriptase. RT was carried out at 25°C for 10 min, 37°C for 120 min, and 85°C for 5 min. After RT, real-time PCR was performed with the TaqMan Gene Expression Master Mix (Applied Biosystems) and run on an ABI Prism 7900HT sequence detection system (Applied Biosystems). After an initial denaturation at 50°C for 2 min and at 95°C for 10 min, 40 cycles of 15 sec at 95°C and 1 min at 60°C were performed. Each subunit mRNA expression level of xCT or 4F2hc was analyzed by the ΔCt method using GAPDH as an endogenous control gene, and the expression level of one sample was determined as the mean value of triplicate determinations.

### 5. Determination of GSH in PC12 cells

PC12 cells (50,000 cells/mL medium) were grown on 100-mm culture dishes and treated with glutamate or glutamate+test substance for 24 h. The cells were scraped off the dishes using a rubber policeman and collected by centrifugation. The cells were suspended in 125 µL PBS, and an equal volume of the MPA reagent was added. The mixture was incubated at room temperature for 5 min and centrifuged at 8,000×*g* for 10 min. The supernatant was mixed with TEAM reagent, and the GSH level in the supernatant was measured using a GSH assay kit based on the glutathione reductase enzymatic recycling method [25], [26] according to the manufacturer's instructions (Cayman Chemical, Ann Arbor, MI, USA). The protein concentration was measured by the method of Lowry et al. [27] with bovine serum albumin as the standard. GSH levels were normalized to cellular protein, and then the levels were expressed as a percentage of the control.

### 6. Statistical analysis

All data were presented as the mean ± S.E.M. The statistical significance of data was assessed by one-way or two-way analysis of variance (ANOVA) followed by Dunnett's or Bonferroni's post hoc test. The significance level in each statistical analysis was accepted at *P*<0.05.

## Results

### Effects of YKS on glutamate-induced PC12 cell death

**Figure 1:**
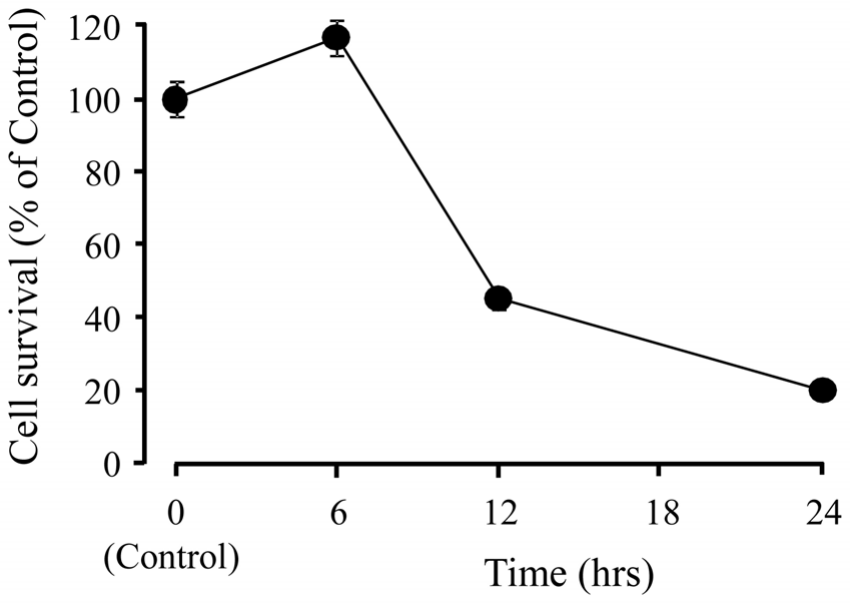
Time-dependent changes in PC12 cell death induced by glutamate. Changes in cell survival rate after the addition of 2.5 mM glutamate to the medium are calculated as percentage of control. Control did not contain glutamate. Each data represents the mean ± S.E.M (n = 6).

**Figure 2:**
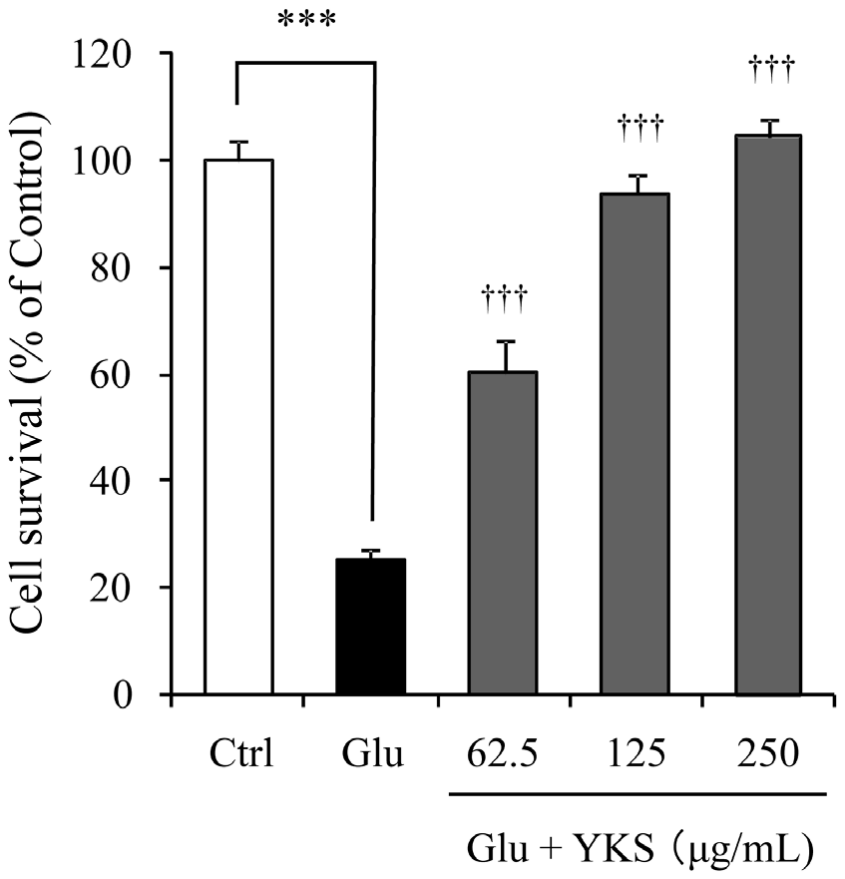
Protective effect of yokukansan on PC12 cell death induced by glutamate. Survival rates in 2.5 mM glutamate (Glu) and Glu+various concentrations (62.5, 125, and 250 µg/mL) of yokukansan (YKS)-treated groups were evaluated 24 h after the addition of Glu to the medium. Control (Ctrl) did not contain glutamate. Each data calculated as a percentage of control represents as the mean ± S.E.M. (n = 6). ^***^ *P*<0.001 vs. Ctrl and ^†††^ *P*<0.001 vs. Glu: one-way ANOVA+Dunnett's test.

Fig. 1 shows the time-dependent changes in PC12 cell death induced by 2.5 mM glutamate. The glutamate did not induce cell death for at least 6 h after it was added to the medium. However, approximately 80% cell death was observed 24 h after glutamate-treatment. YKS (62.5–250 µg/mL) prevented the glutamate-induced PC12 cell death at 24 h in a concentration-dependent manner (Fig. 2). Treating PC12 cells with YKS alone had no significant effect (data not shown).

**Figure 3:**
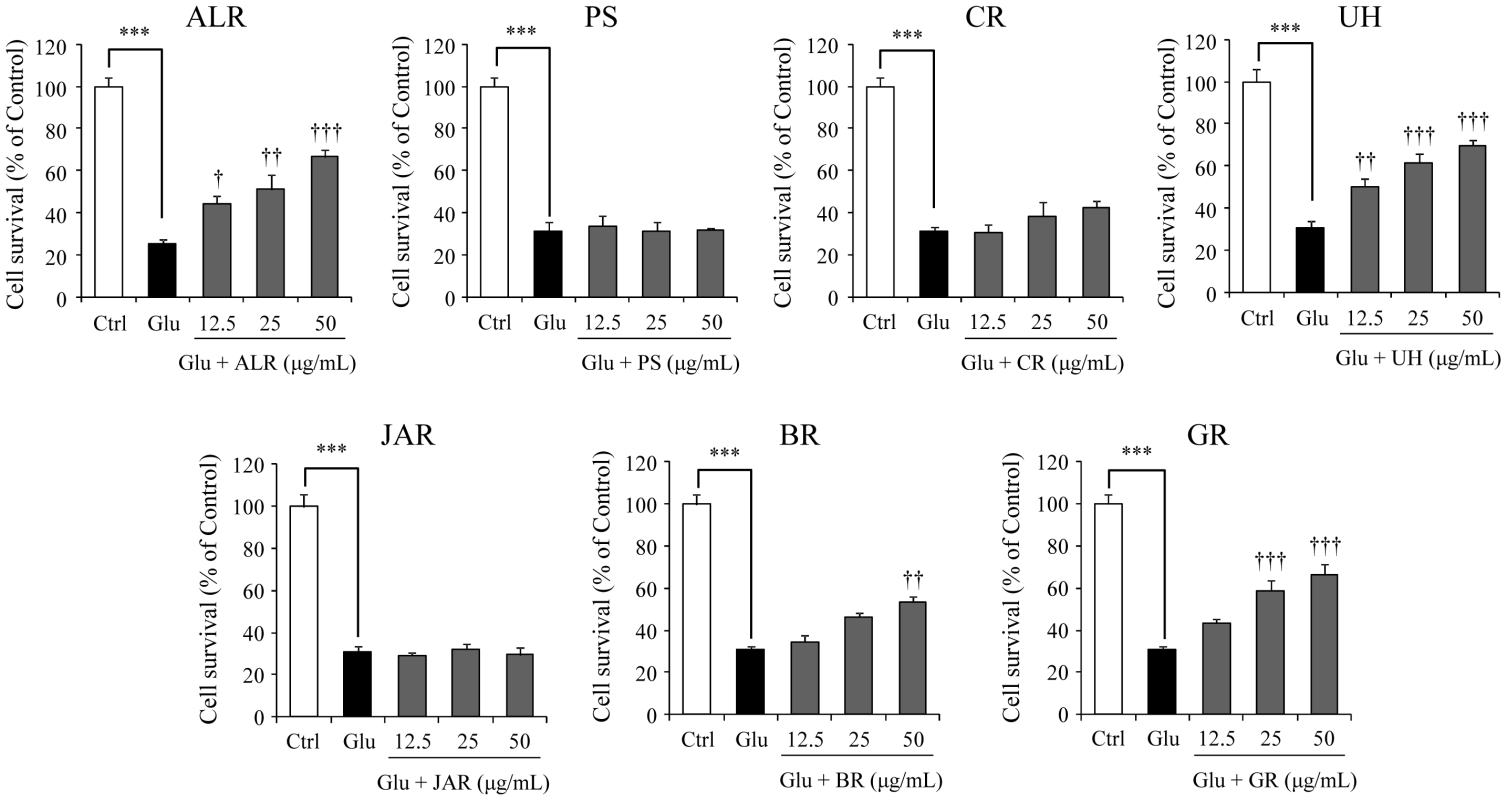
Effects of seven constituent herbs of yokukansan on glutamate-induced PC12 cell death. Survival rates in 2.5 mM glutamate (Glu) and Glu+various concentrations (12.5, 25 and 50 µg/mL) of seven constituent herb-treated groups were evaluated 24 h after addition of Glu to the medium. Abbreviations: Atractylodes Lancea rhizome (ALR), Poria sclerotium (PS), Cnidium rhizome (CR), Uncaria hook (UH), Japanese Angelica root (JAR), Bupleurum root (BR), and Glycyrrhiza (GR). Control (Ctrl) did not contain glutamate. Each data calculated as a percentage of the corresponding control represents as the mean ± S.E.M. (n = 6). ^***^ *P*<0.001 vs. Ctrl, ^†^ *P*<0.05, ^††^ *P*<0.01 and ^†††^ *P*<0.001 vs. Glu: one-way ANOVA+Dunnett's test.

Fig. 3 shows the effects of seven constituent herbs (12.5, 25, and 50 µg/mL) on the cell death of PC12 cells treated with 2.5 mM glutamate for 24 h. Higher inhibition potency was found for ALR, UH, and GR. BR significantly inhibited glutamate-induced cell death only at high concentration (50 µg/ml). Table 1 shows the correlative formula between concentration and inhibition rate of each constituent herb against 2.5 mM glutamate-induced cell death. From these formulas, the fifty percent inhibitory concentration (IC_50_) was calculated to be 42.40, 38.45, and 43.86 µg/mL for ALR, UH, and GR, respectively. The IC_50_ values of other constituents including BR were not detected within a range of experimental concentration (12.5–50 µg/mL).

**Table 1:** Correlative formulas between concentrations and inhibition rates of seven constituent herbs against 2.5 mM glutamate-induced cell death.

Constituent herbs	R^2^	Regression equationa)	IC_50_ (µg/mL)
Atractylodes Lancea Rhizome	0.9423	y = 1.0377x+5.997	42.40
Poria Sclerotium	0.0035	y = 0.0043x+1.1158	ND
Cnidium Rhizome	0.8739	y = 0.3611x−1.4684	ND
Uncaria Hook	0.8728	y = 1.0696x+8.8713	38.45
Japanese Angelica Root	0.0358	y = −0.0158x−0.2283	ND
Bupleurum Root	0.9377	y = 0.6797x+0.2688	ND
Glycyrrhiza	0.9090	y = 1.0246x+5.0594	43.86

R^2^: correlation co-efficient.

a) y: % of amelioration, x: the amount of constituent herb in µg/mL.

ND: IC_50_ was not determined within a range of experimental concentrations (12.5–50 µg/mL).

**Figure 4:**
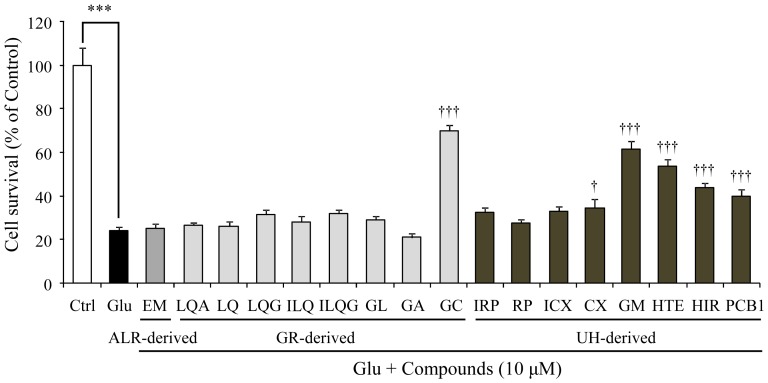
Effects of 17 Atractylodes Lancea rhizome-, Glycyrrhiza-, and Uncaria hook-derived components on glutamate-induced PC12 cell death. Survival rates in groups treated with 2.5 mM glutamate (Glu) or Glu+various components were evaluated 24 h after the addition of Glu to the medium at a fixed concentration of 10 µM. Control (Ctrl) did not contain glutamate. Abbreviations: Atractylodes Lancea rhizome (ALR), Glycyrrhiza (GR), Uncaria hook (UH), β-Eudesmol (EM), liquiritinapioside (LQA), liquiritin (LQ), liquiritigenin (LQG), isoliquiritin (ILQ), isoliquiritigenin (ILQG), glycyrrhizic acid (GL), 18β-glycyrrhetinic acid (GA), glycycoumarin (GC), isorhynchophylline (IRP), rhynchophylline (RP), isocorynoxeine (ICX), corynoxeine (CX), geissoschizine methyl ether (GM), hirsuteine (HTE), hirsutine (HIR), and procyanidin B1 (PCB1). Each data calculated as a percentage of the control represents as the mean ± S.E.M. (n = 6). ^***^ *P*<0.001 vs. Ctrl, and ^†^ *P*<0.05 and ^†††^ *P*<0.001 vs. Glu: one-way ANOVA+Dunnett's test.

**Figure 5:**
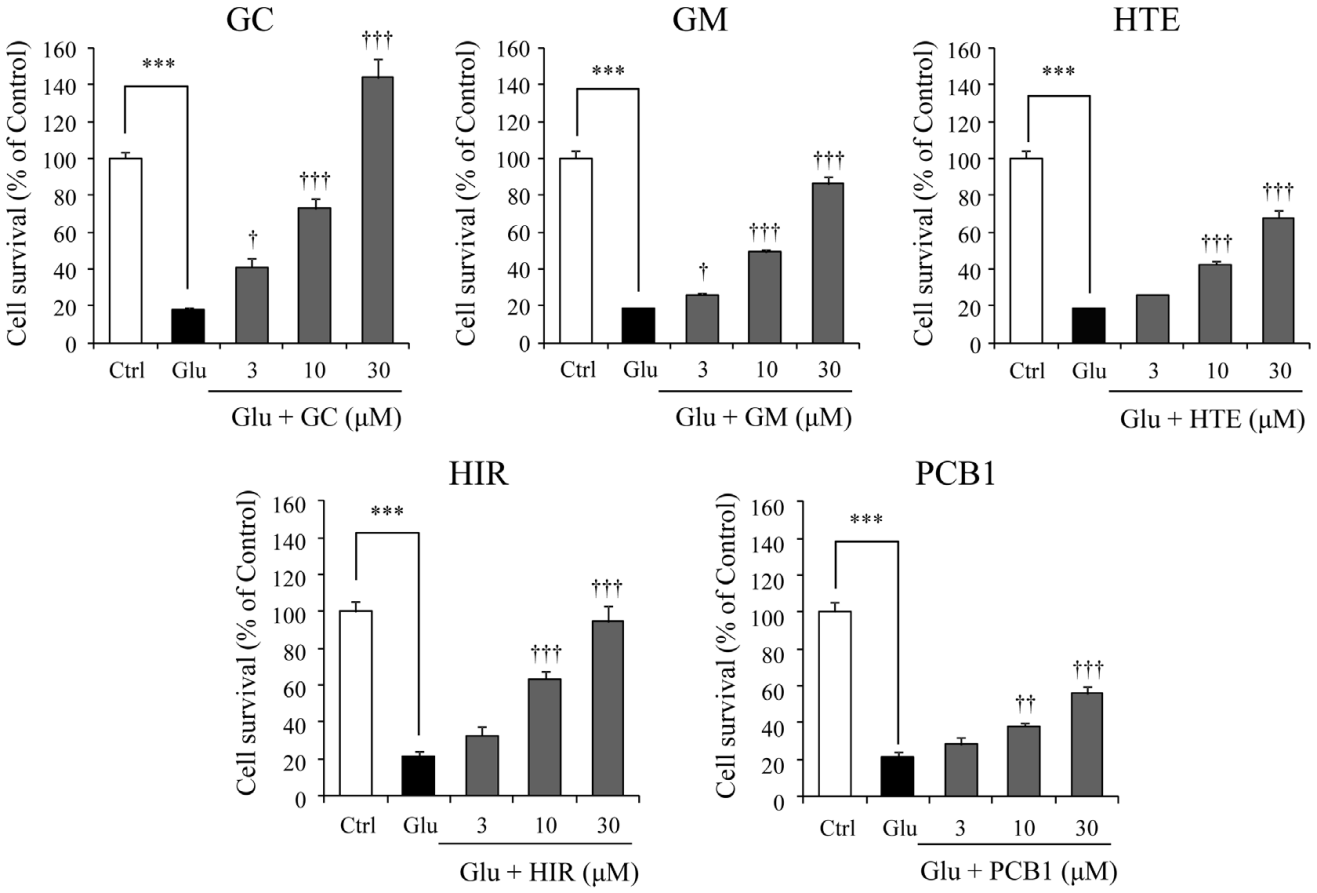
Concentration-dependent protective effects of glycycoumarin, geissoschizine methyl ether, hirsuteine, hirsutine, and procyanidin B1 on glutamate-induced PC12 cell death. Survival rates in 2.5 mM glutamate (Glu) and Glu+each ingredient (3, 10, and 30 µM)-treated group were evaluated 24 h after the addition of Glu to the medium. Control (Ctrl) did not contain glutamate. Abbreviations: glycycoumarin (GC), geissoschizine methyl ether (GM), hirsuteine (HTE), hirsutine (HIR), and procyanidin B1 (PCB1). Each data calculated as a percentage of the control represents the mean ± S.E.M. (n = 6). ^***^ *P*<0.001 vs. Ctrl, and ^†^ *P*<0.05, ^††^ *P*<0.01, and ^†††^ *P*<0.001 vs. Glu: one-way ANOVA+Dunnett's test.

We prepared 17 components including one ALR-, eight GR-, and eight UH-derived components, and the effects of these ingredients on the glutamate-induced cell death were examined at a fixed concentration of 10 µM (Fig. 4). Significant inhibitory effects against glutamate-induced cell death were found for GC in GR and GM, HTE, HIR, and PCB1 in UH. A concentration-dependent inhibition response (3, 10, and 30 µM) was observed in these effects (Fig. 5). Table 2 shows the correlative formula between concentration and inhibition rate of each constituent against 2.5 mM glutamate-induced cell death. From these formulas, the IC_50_ values were calculated to be 8.27, 17.07, 23.64, 14.02, and 33.59 µM for GC, GM, HTE, HIR, and PCB1, respectively.

**Table 2:** Correlative formulas between concentrations and inhibition rates of five UH-derived components against 2.5 mM glutamate-induced cell death.

Components	R^2^	Regression equationa)	IC_50_ (µM)
Glycycoumarin	0.9856	y = 4.8994x+9.4969	8.27
Geissoschizine methyl ether	0.9828	y = 2.7497x+3.0542	17.07
Hirsuteine	0.9744	y = 1.9605x+3.6496	23.64
Hirsutine	0.9365	y = 3.0083x+7.8112	14.02
Procyanidin B1	0.9709	y = 1.384x+3.515	33.59

R^2^: correlation co-efficient.

a) y: % of amelioration, x: the amount of component in µM.

### Effects of YKS on mRNA expressions of system Xc^−^ subunits xCT and 4F2hc

**Figure 6:**
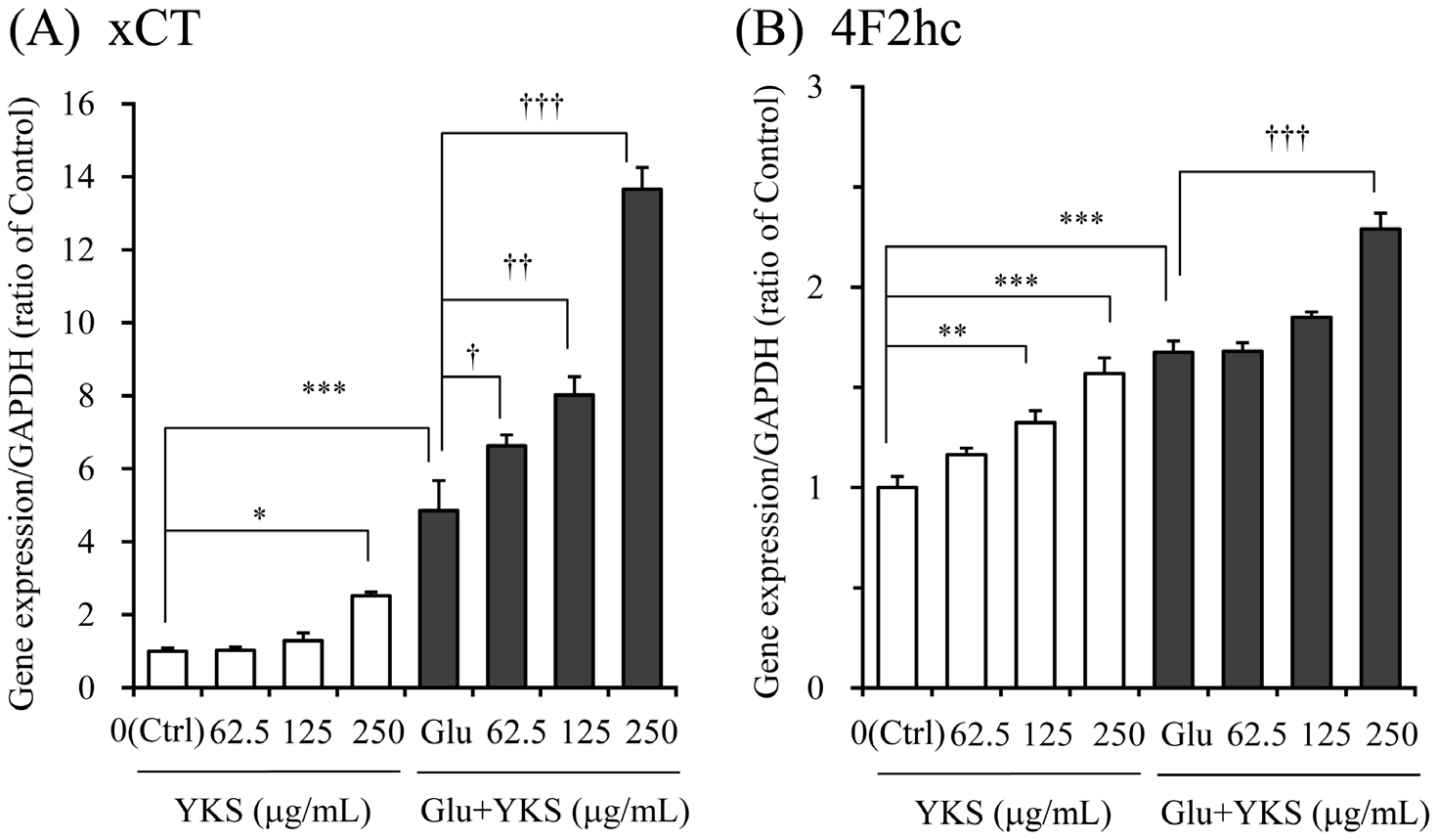
Effects of yokukansan on gene expressions of system Xc^−^ subunits xCT (A) and 4F2hc (B) in PC12 cells. The effects of yokukansan (YKS: 62.5, 125, and 250 µg/mL) was examined 6 h after incubation under the conditions with or without 2.5 mM glutamate (Glu). Control (Ctrl) did not contain glutamate. The gene expression level in each sample was determined as the mean value of triplicate determinations. Each data calculated as the ratio of endogenous control gene GAPDH represents as the mean ± S.E.M. (n = 3). ^*^ *P*<0.05, ^**^ *P*<0.01 and ^***^ *P*<0.001 vs. Ctrl, and ^†^ *P*<0.05, ^††^ *P*<0.01, and ^†††^ *P*<0.001 vs. Glu: two-way ANOVA+Dunnett's test.

The effects of YKS (62.5–250 µg/mL) on gene expressions of the system Xc^−^ subunits xCT (Fig. 6A) and 4F2hc (Fig. 6B) were evaluated 6 h after the addition of saline or 2.5 mM glutamate to the medium. The statistical significance on group factor (G_f_) or concentration factor (C_f_) was observed in both gene expressions of xCT (G_f_: F_1,16_ = 516.8, P<0.001, C_f_: F_3,16_ = 56.4, P<0.001) and 4F2hc (G_f_: F_1,16_ = 228.6, P<0.001, C_f_: F_3,16_ = 42.1, P<0.001). Post hoc analysis showed that YKS increased the mRNA expressions of both xCT and 4F2hc in the non-glutamate (saline)-treated condition. In the glutamate-treated condition, the gene expressions of both subunits by 2.5 mM glutamate were significantly increased compared to those of controls. Co-treatment with YKS and glutamate further augmented the gene expressions of both subunits.

**Figure 7:**
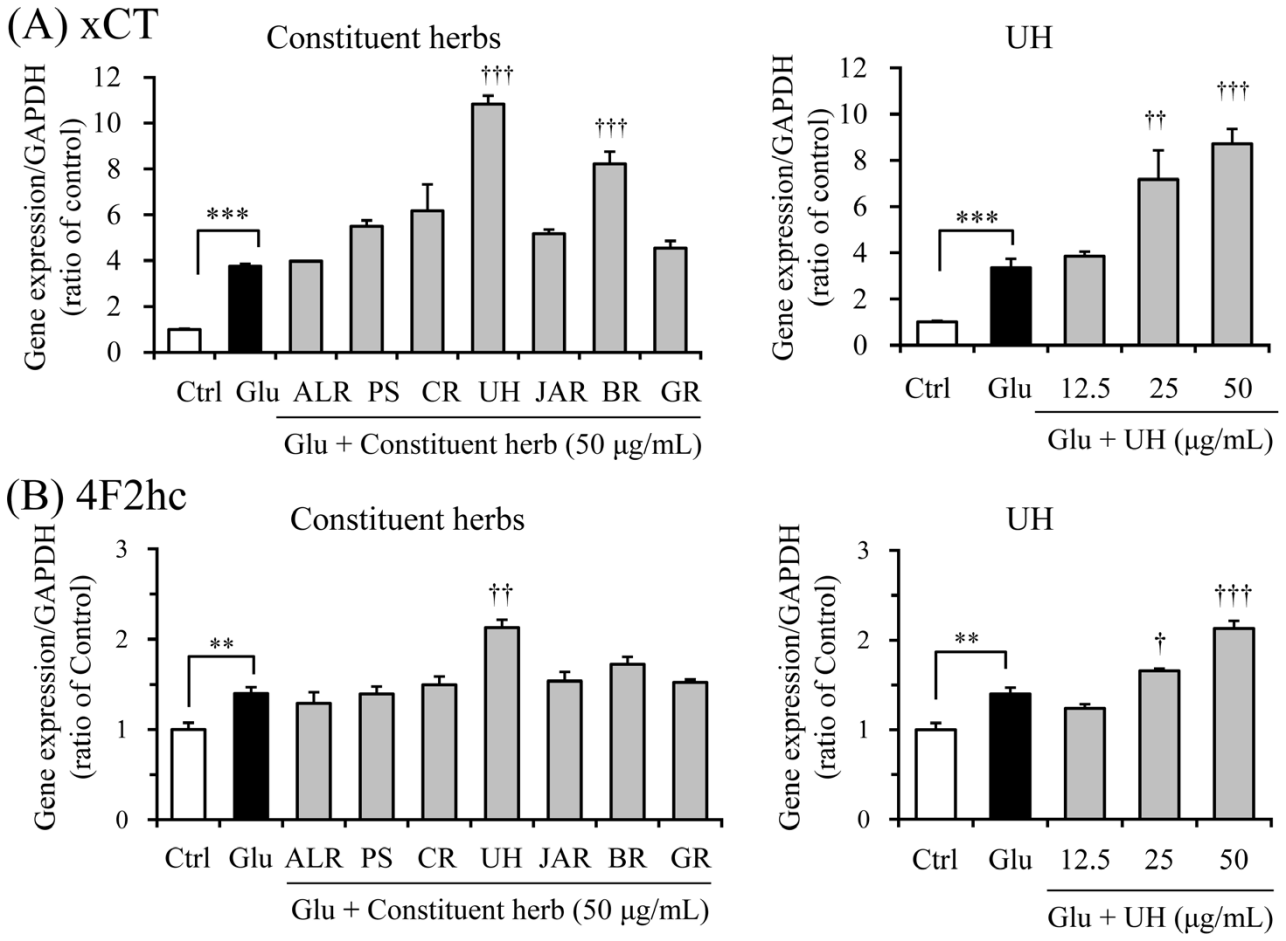
Screening test for detection of active herbs facilitating gene expression of xCT (A) and 4F2hc (B). The effects of seven constituent herbs (a fixed concentration of 50 µM) and UH (12.5, 25, and 50 µg/mL) were examined 6 h after incubation under the conditions without or with 2.5 mM glutamate (Glu). The gene expression level in one sample was determined as the mean value of triplicate determinations. Control (Ctrl) did not contain glutamate. Abbreviations: Atractylodes Lancea rhizome (ALR), Poria sclerotium (PS), Cnidium rhizome (CR), Uncaria hook (UH), Japanese Angelica root (JAR), Bupleurum root (BR), and Glycyrrhiza (GR). Each data calculated as the ratio of endogenous control gene GAPDH represents the mean ± S.E.M. (n = 3). ^***^ *P*<0.001 vs. Ctrl, and ^†^ *P*<0.05, ^††^ *P*<0.01, and ^†††^ *P*<0.001 vs. Glu: one-way ANOVA+Dunnett's test.

The active constituent herbs were screened at a fixed concentration of 50 µg/mL (Fig. 7). Glutamate treatment significantly increased the gene expressions of both subunits compared to those of controls. Gene expression effects similar to that of YKS against xCT (Fig. 7A) and 4F2hc (Fig. 7B) were found in UH. The concentration dependency was observed in both gene expressions of UH (Fig. 7A and Fig. 7B). BR increased xCT gene expression but not 4F2hc gene expression.

**Figure 8:**
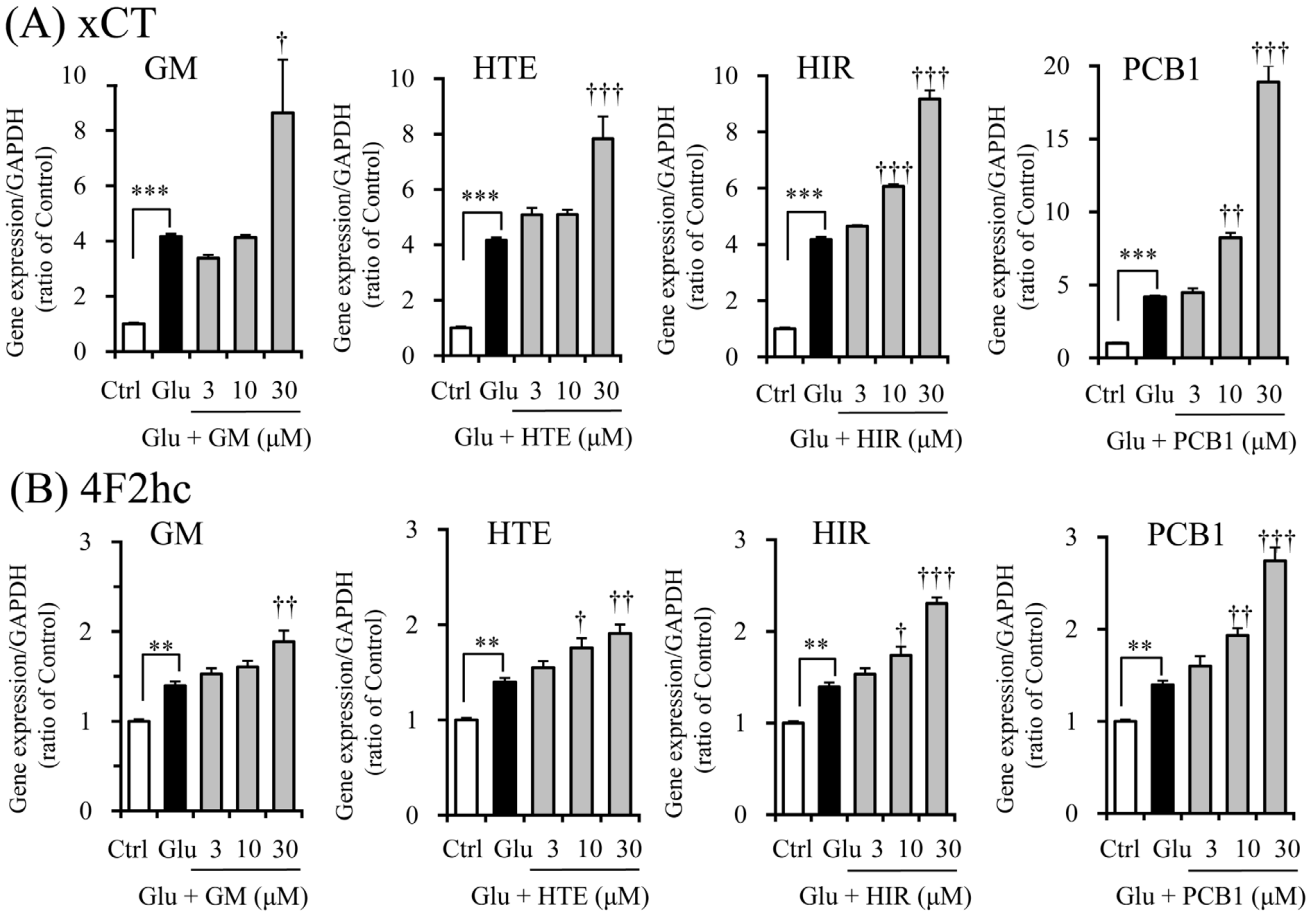
Effects of geissoschizine methyl ether, hirsuteine, hirsutine and procyanidin B1 on gene expressions of system Xc^−^ subunits xCT (A) and 4F2hc (B) in PC12 cells. The effects of each component (3, 10, and 30 µM) were examined 6 h after incubation under the conditions without or with 2.5 mM glutamate (Glu). The gene expression level in one sample was determined as the mean value of triplicate determinations. Control (Ctrl) did not contain glutamate. Abbreviations: glycycoumarin (GC), geissoschizine methyl ether (GM), hirsuteine (HTE), hirsutine (HIR), and procyanidin B1 (PCB1). Each data calculated as the ratio of endogenous control gene GAPDH represents the mean ± S.E.M. (n = 3). ^**^ *P*<0.01 and ^***^ *P*<0.001 vs. Ctrl, and ^†^ *P*<0.05, ^††^ *P*<0.01 and ^†††^ *P*<0.001 vs. Glu: one-way ANOVA+Dunnett's test.

Next, we examined the effects of four UH-derived components, GM, HTE, HIR, and PCB1, that had demonstrated cytoprotection (Fig. 5) on both gene expressions (Fig. 8A and Fig. 8B). GM, HTE, HIR, and PCB1 further enhanced glutamate-induced increases in the gene expressions of both subunits.

### Effect of YKS on GSH level in PC12 cells

**Figure 9:**
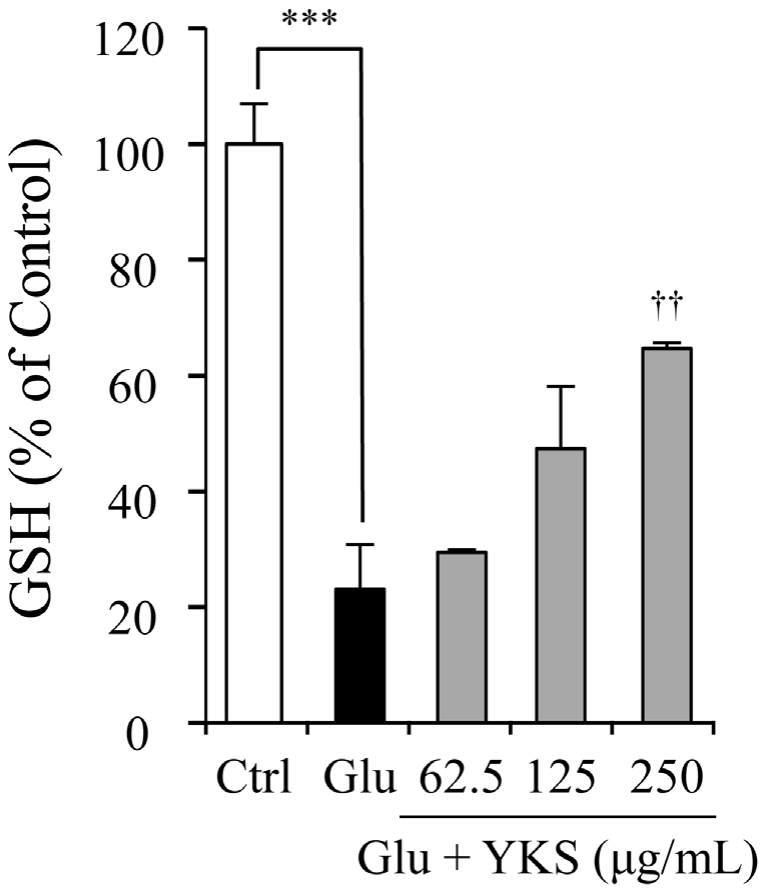
Ameliorative effect of yokukansan on glutamate-induced decrease in GSH levels in PC12 cells. The GSH levels in 2.5 mM glutamate (Glu) and Glu+yokukansan (YKS: 62.5, 125, and 250 µg/mL)-treated groups were measured 24 h after the addition of Glu to the medium. Control (Ctrl) did not contain glutamate. Each data calculated as a percentage of the control represents the mean ± S.E.M. (n = 6). ^***^ *P*<0.001 vs. Ctrl and ^††^ *P*<0.01 vs. Glu: one-way ANOVA+Dunnett's test.

**Figure 10:**
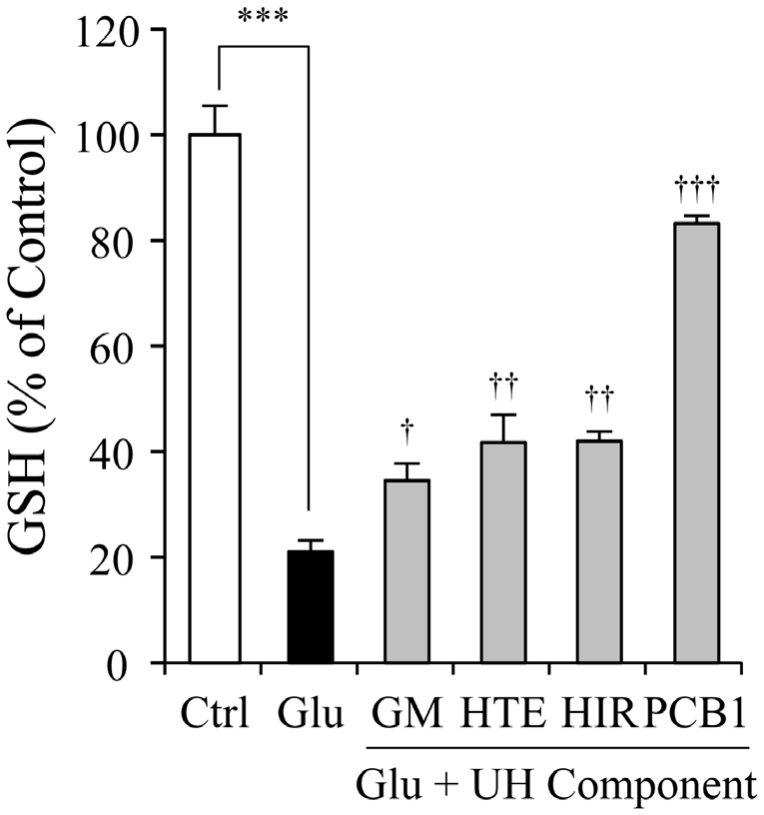
Ameliorative effects of geissoschizine methyl ether, hirsuteine, hirsutine, and procyanidin B1 on glutamate-induced decrease in GSH levels in PC12 cells. The GSH levels in 2.5 mM glutamate (Glu) and Glu+each ingredient (30 µM)-treated groups were measured 24 h after the addition of Glu to the medium. Control (Ctrl) did not contain glutamate. Abbreviations: glycycoumarin (GC), geissoschizine methyl ether (GM), hirsuteine (HTE), hirsutine (HIR), and procyanidin B1 (PCB1). Each data calculated as a percentage of the control represents the mean ± S.E.M. (n = 6). ^***^ *P*<0.001 vs. Ctrl, and ^†^ *P*<0.05, ^††^ *P*<0.01, and ^†††^ *P*<0.001 vs. Glu: one-way ANOVA+Dunnett's test.

Fig. 9 shows the effects of YKS (62.5, 125, and 250 µg/mL) on GSH levels in PC12 cells treated with 2.5 mM glutamate for 24 h. The glutamate decreased the GSH level up to 20% of the control. YKS ameliorated the decrease in GSH level in a concentration-dependent manner. Fig. 10 shows the effects of four UH-derived components (GM, HTE, HIR, and PCB1 that induced gene expressions of xCT and 4F2hc) on GSH. These components significantly ameliorated the glutamate-induced decrease in GSH.

### Effects of glycycoumarin on mRNA expressions of system Xc^−^ subunits and GSH level in PC12 cells

**Figure 11:**
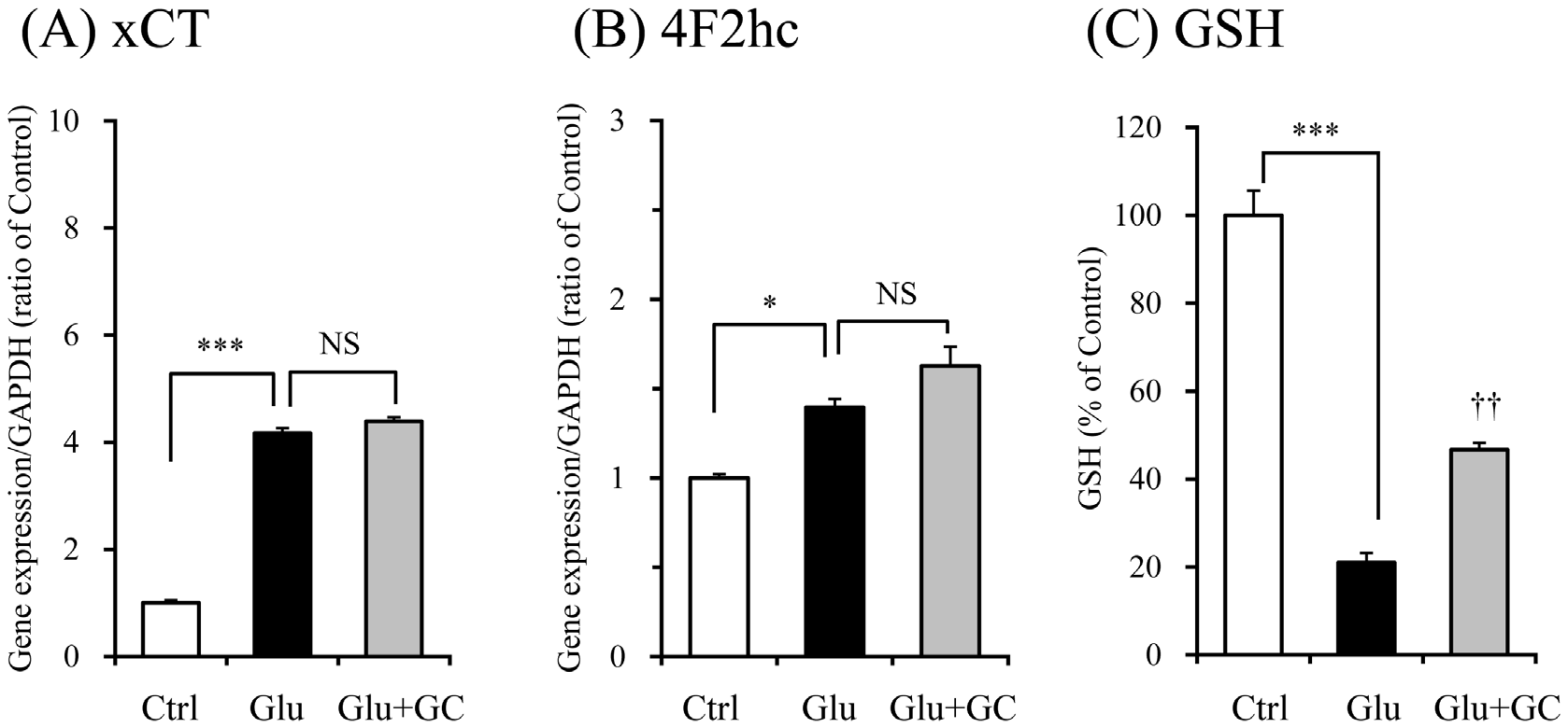
Effects of glycycoumarin on mRNA expressions of system Xc^−^ subunits and GSH level in PC12 cells. The effects of glycycoumarin (30 µM) on gene expressions of xCT (A) and 4F2hc (B) were examined 6 h after incubation under the conditions without or with 2.5 mM glutamate (Glu). Control (Ctrl) did not contain glutamate. The gene expression level in one sample was determined as the mean value of triplicate determinations. The GSH level (C) in each group was measured 24 h after the addition of Glu to the medium. Each data calculated as a percentage of the control represents the mean ± S.E.M. (n = 6). ^*^ *P*<0.05 and ^***^ *P*<0.001 vs. Ctrl, and ^††^ *P*<0.01 vs. Glu: one-way ANOVA+Dunnett's test. NS: non-significant.

Both gene expressions of xCT (Fig. 11A) and 4F2hc (Fig. 11B) were significantly increased by glutamate. These increases were not affected at all by 30 µM glycycoumarin, which showed strong cytoprotective effect (Fig. 5). However, GSH level was significantly increased by this component (Fig. 11C).

### Inhibitory effects of CPG on protective effects of YKS and four UH-derived components on glutamate-induced PC12 cell death

**Figure 12:**
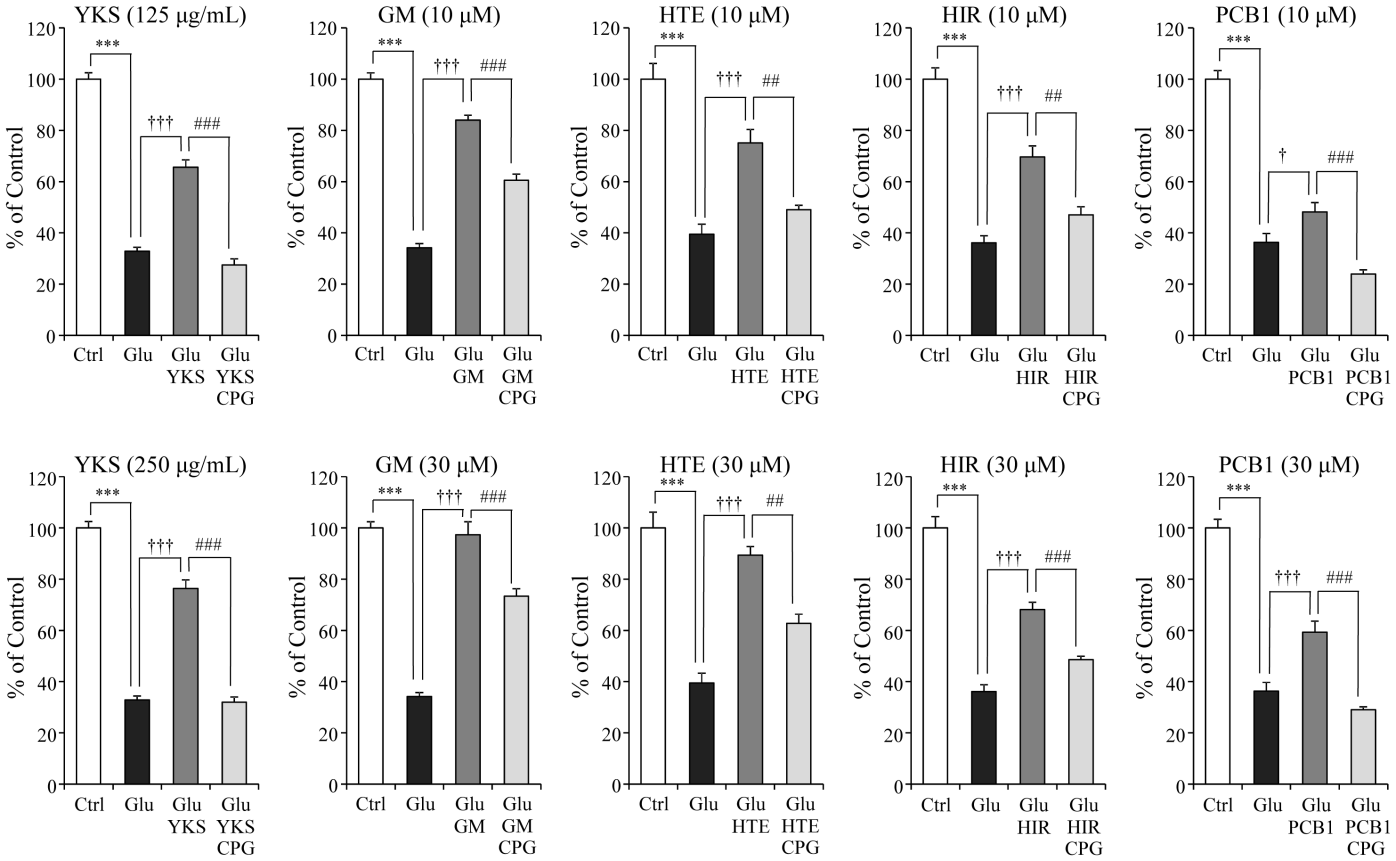
Inhibitory effects of CPG on protective effects of YKS and four UH-derived components on glutamate-induced PC12 cell death. Survival rates in 2.5 mM glutamate (Glu), Glu+125 or 250 µg/mL yokukansan (YKS), Glu+10 or 30 µM UH alkaloid (geissoschizine methyl ether: GM, hirsuteine: HTE, hirsutine: HIR, and procyanidin B1: PCB1) or Glu+10 or 30 µM test substance+100 or 300 µM CPG-treated groups were evaluated 24 h after the addition of Glu to the medium. Control (Ctrl) did not contain glutamate. Each data calculated as a percentage of control represents as the mean ± S.E.M. (n = 6). ^***^ *P*<0.001 vs. Ctrl, ^†^ *P*<0.05 and ^†††^ *P*<0.001 vs. Glu, and ^##^ *P*<0.01 and ^###^ *P*<0.001 vs. each test substance: one-way ANOVA+Bonferroni's test.

In other set of experiment, we examined the inhibitory effect of a system Xc^−^ inhibitor, CPG, on cytoprotection of YKS and four UH-derived components (GM, HTE, HIR, and PCB1) on glutamate-induced PC12 cell death to verify whether these cytoprotective effects are related to system Xc^−^ (Fig. 12). Glutamate (2.5 mM) significantly induced cell death. The cell death was significantly ameliorated by YKS (125 and 250 µg/mL), GM (10 and 30 µM), HTE (10 and 30 µM), HIR (10 and 30 µM) and PCB1 (10 and 30 µM), respectively. The cytoprotective effects of 125 µg/mL YKS and 10 µM component were significantly inhibited by co-treatment with 100 µM CPG. The effects of 250 µg/mL YKS and 30 µM component were significantly inhibited by 300 µM CPG.

## Discussion

The cystine/glutamate antiporter system Xc^−^ imports the amino acid cystine, the oxidized form of cysteine, into cells with a 1∶1 counter-transport of glutamate. It consists of two protein components, the 4F2 heavy chain (4F2hc), necessary for membrane location of the heterodimer, and the xCT protein, responsible for transporter activity. Cysteine is the rate-limiting substrate for the antioxidant glutathione (GSH), along with cystine [13], [28]–[30]. PC12 cells are demonstrated to express the system Xc^−^ subunits but not a normal NMDA receptor profile [10]–[14]. We also previously confirmed the absence of NMDA receptor subunits NR2A and NR2B in PC12 cells, although system Xc^−^ subunits xCT and 4F2hc are expressed in PC12 cells as well as neurons as revealed by real-time RT-PCR analysis [15]. These findings suggest that the PC12 cell is a valuable tool for selective evaluation of the effects of test substances on system Xc^−^.

YKS inhibited glutamate-induced PC12 cell death. We clarified that ALR, UH, BR, and GR were responsible for this effect of YKS. The IC_50_ values were 42.40, 38.45, and 43.86 µg/mL for ALR, UH, and GR, respectively. However, the IC_50_ values of other constituents including BR were not detected within a range of experimental concentration (12.5–50 µg/mL). From this result, we judged that the main crude drugs which participated in the cytoprotective effect of YKS were ALR, UH, and GR. In vitro study using crude extract such as YKS and its constituent herb is an experiment in an unreal environment. However, this procedure is an appropriate screening to find out active components from crude extract. In other words, the results demonstrated that YKS and its constituent herb had pharmacological activity suggest that active components are included in those crude extract. By using this procedure, we previously demonstrated that UH-derived GM, HTE, and HIR were responsible for the cytoprotective effect of YKS [15]. In addition to these components, in the present study, GR-derived GC and UH-derived PCB1 were newly found to be active components in the cytoprotection. Unfortunately, we could not clarify the active components in the case of ALR because it was impossible to prepare adequate number of the components. However, the cytoprotection data of ALR shown in Fig. 3 suggests a possibility that ALR contains active components, although a detailed examination will be necessary in the future.

Because the synthesis of antioxidant GSH for cytoprotection is facilitated by activation of system Xc^−^ [10], [29], the cytoprotective effects of YKS, its constituent herbs, and their components are inferred to be closely related to system Xc^−^. To clarify this hypothesis, gene expressions of xCT and 4F2hc were analyzed in an early stage in the process of glutamate-induced cell death. Glutamate exposure for 6 h against PC12 cells induced significant increase in the expression of xCT and 4F2hc, and cell death was not observed at all. However, the exposure to glutamate for 24 h induced cell death. From these results, we inferred that the cell survival against 6 hour-exposure of glutamate may be due to the defense by over expressing system Xc^−^ genes, and the cell death caused by 24 hour-exposure of glutamate may be due to broke-down of this defense. YKS protected from glutamate-induced cell death by augmenting gene expression of both xCT and 4F2hc. UH showed a similar effect, but none of the other constituent herbs showed the effect. From these results, we suggest that the cytoprotective effect of UH might be related to activation of system Xc^−^ and also that the active components are contained in UH.

We previously demonstrated that the elimination of cystine from the culture medium decreased GSH level in PC12 cells and induced the cell death [15]. In addition, CPG, a cystine/glutamate antiporter inhibitor, also decreased GSH level and induced cell death [31]. YKS inhibited the cell death induced by cystine deficiency or CPG [15]. These results suggest that YKS protects cell death by directly increasing intracellular GSH level also in the abeyance of the antiporter. For example, GSH is demonstrated to be increased via the Nrf2-dependent induction of the GSH synthesis [32]. In this previous study, we suggested that the anti-oxidative effect of YKS may be attributed to GM, HTE and HIR in UH, because these components ameliorated glutamate-induced decrease in GSH levels and protected cell death [15]. However, the effects of YKS or components on the system Xc^−^ remain unclear. In the present study, we newly demonstrated that GM, HTE, HIR, and PCB1 had a protective effect against glutamate-induced cell death. In addition, these four components enhanced gene expressions of xCT and 4F2hc, and also ameliorated a glutamate-induced decrease in GSH levels. In the four components, however, PCB1 increased both xCT and 4F2hc gene expressions and GSH level compared with other three components, but the cell protective effect was lesser than other three components. Although this discrepancy is not cleared in this study, the experiment using system Xc^−^ inhibitor showed that cytoprotective effects of YKS and four UH-derived components (GM, HTE, HIR, and PCB1) were partly counteracted by co-treatment with system Xc^−^ inhibitor (Fig. 12). These results suggest that the enhancement of system Xc^−^ gene expression by GM, HTE, HIR or PCB1 may contribute to at least part of the cytoprotective effect of YKS. Taken together with the present study and previous study [15], the cytoprotection by YKS is thought to be composed of two different mechanisms: the protection by directly increasing intracellular GSH level (e.g., the Nrf2-dependent GSH production) and the protection by enhancement of system Xc^−^. GM, HTE, HIR, and PCB1 may be included in the group having two different mechanisms. On the other hand, glycycoumarin showed a more potent cytoprotective effect with significant increase in GSH level. However, this component had no effect on the expression of xCT or 4F2hc gene (Fig. 11A and B). Therefore, glycycoumarin is thought to be a component which directly activates GSH not via system Xc^−^. The present study was performed using PC12 cells. In order to ensure that the present results are not cell line specific, it is necessary to verify the present results by using the other cell line in the future.

As shown in Fig. 3, ALR, BR, and GR protected glutamate-induced cell death without mediation of system Xc^−^ (xCT and 4F2hc). Recently, Kubota et al. [33] have demonstrated in PC12 cells that YKS induces NGF-like phosphorylation and activation of protein kinase and lipid kinase pathways including extracellular signal-regulated kinase 1/2 (ERK1/2) and phosphatidylinositol 3-kinase/Akt (PI3K/Akt), which are known to regulate neuronal protection, proliferation, and differentiation [34], [35] and neurite outgrowth [36]. YKS has also been demonstrated to suppress Aβ-induced neuronal apoptosis through the suppression of caspase-3 activation [37]. Thus, activation of ERK1/2 and PI3K/Akt in PC12 cells may be involved in the cytoprotection of YKS.

Cognitive dysfunction and behavioral and psychological symptoms of dementia are thought to be associated with neurofunctional and neuropathological abnormalities in the brain. In several animal models used to study the pathogenesis and therapy of demantia, an increase in the extracellular levels of excitatory amino acids such as glutamate in the brain has been demonstrated [38]–[41]. Glutamate is well-known to contribute not only to induction of excitation of post-synaptic neurons but also to excitotoxic neuronal death due to the intensity and duration of glutamate exposure [6], [42]. We previously demonstrated that YKS ameliorated thiamine deficiency-induced degeneration of neuronal and astroglial cells in the rat brain stem, cerebral cortex, and hippocampus, which are responsible for learning, memory, and various psychological functions [43], [44]. YKS also ameliorated glutamate cytotoxicity by reducing dysfunction of glutamate uptake in astrocytes [21]. It is thought that GA, a metabolite of GL, is likely to be responsible as an active component for glutamate transport [22]. The activation of glutamate transport in addition to system Xc^−^ is, at least, thought to participate in protective effects of YKS against glutamate-induced cytotoxicity.

We previously demonstrated that GM, HTE and HIR were detected in the plasma and brain of rats orally administered YKS [45]. These findings suggest that these components in orally administered YKS are absorbed into the blood, and then reach the brain through the blood-brain barrier.

In conclusion, the present study is the first demonstrating that YKS protects glutamate cytotoxicity by augmenting gene expressions of system Xc^−^ subunits, 4F2hc and xCT. UH-derived GM, HTE, HIR, and PCB1 are, at least, thought to be responsible for the cytoprotective effect of YKS.
